# Chronic Obstructive Pulmonary Disease in Latin America

**DOI:** 10.5334/aogh.2418

**Published:** 2019-01-22

**Authors:** Rogelio Perez-Padilla, Ana Maria B. Menezes

**Affiliations:** 1 Instituto Nacional de Enfermedades Respiratorias, Ciudad de México, MX; 2 Federal University of Pelotas, BR

## Abstract

The PLATINO and PREPOCOL population-based studies documented the prevalence of chronic obstructive pulmonary disease (COPD) in several Latin American (Mexico City, Sao Paulo, Montevideo, Santiago and Caracas) and Colombian (Medellin, Bogota, Barranquilla, Bucaramanga and Cali) cities. COPD ranged between 6.2 and 19.6% in individuals ≥40 years of age, with substantial rates of underdiagnosis (up to 89%) but also overdiagnosis, mostly due to the lack of spirometric confirmation. The main risk factor was tobacco smoking, but male gender and age were also associated with COPD. COPD in never smokers represented about one third of the cases and was associated with previous history of tuberculosis or a diagnosis of asthma. COPD associated with biomass smoke exposure was a common clinical phenotype in Latin America, found as a risk factor in PREPOCOL and other observational studies in the region. Smoking has been decreasing in Latin America and efforts have been made to implement cleaner biomass stoves. Unfortunately, treatment of COPD in Latin America remains highly variable with low rates of smoking cessation counselling, low use of inhaled bronchodilators and influenza vaccination. A primary-care approach to COPD, particularly in the form of integrated programs is lacking but would be critical to improving rates of diagnosis and treatment of COPD.

## Background

Latin America covers a vast geographic area (larger than the United States, Canada or Western Europe) with about 600 million inhabitants distributed in at least 19 countries. The population tripled since 1950, with a massive migration from rural areas to cities. Latin America is a mixed pot of ethnicities including the European, African, Mulatto, Mestizo and Amerindian individuals, in various proportions depending on the native population prior to the arrival of Europeans and the extent of European and African migration. Unfortunately, Latin America is one of the areas in the world with the widest disparity in income within and between countries, leading to a coexistence of diseases of poverty with those seen in developed countries. Tobacco-induced diseases, diabetes, obesity and the cardiovascular disease typical of developed regions and infectious diseases and problems related to violence often overlap in the same country.

## Prevalence of COPD in Latin America: Data from Population-based Studies

**Figure 1:**
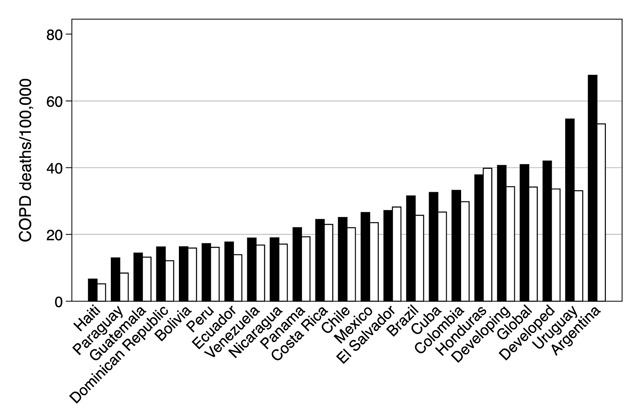
Deaths due to chronic obstructive pulmonary disease per 100,000 habitants according to the 2013 Global Burden of Disease study in men (black bars) and women (grey bars). Includes bars for the world estimate (global) as well as for developed and developing countries.

Before several population-based surveys conducted in the early 2000s, information on respiratory diseases was very scarce, beyond the compulsory data provided by the World Health Organization (WHO), mostly based on mortality statistics. The Global Burden of Disease (GBD) project (the latest conducted in 2013) [1] provided estimates for mortality and disability-adjusted life years (DALY) lost in multiple Latin American countries (Figure 1) [2]. A wide range of mortality in Latin America was observed either expressed as crude or age-standardized rates (not shown). While these data are useful, mortality rates due to COPD does not provide reliable information about the prevalence of this disease due to high levels of under- and overdiagnosis. The first population-based surveys of COPD prevalence in Latin America using spirometry (the standard diagnostic test) [3] were the PLATINO and PREPOCOL studies. PLATINO, designed and supported by the Latin American Thoracic Association (ALAT), provided the first standardized estimates of the prevalence of COPD and other respiratory diseases [4]. This was a population-based survey, conducted in five highly populated urban areas: Mexico City, Mexico; Caracas, Venezuela; Santiago, Chile; Sao Paulo, Brazil; and Montevideo, Uruguay using methods consistent with the international Burden of Obstructive Lung Diseases (BOLD) study. The study provided important information from areas with varied rates of racially mixed individuals as well as subjects of African ancestry, thus, offering a representative picture of the burden of COPD in urban Latin America. A second multicity study, PREPOCOL, was conducted in Colombia including five cities at varied altitudes above sea level [5].

Testing in multicenter studies, such as PLATINO and PREPOCOL, require strict quality control [6] including the use of reliable spirometers with adequate reference values and criteria of airflow obstruction [7]. Although the GOLD Committee defines COPD as a forced expiratory volume in one second (FEV_1_) over forced vital capacity (FVC) ratio < 0.70, worldwide evidence suggests limitations of age-biased criteria, as the ratio in normal subjects decreases with aging. In elders, over diagnosis of COPD can be substantial if a fixed-ratio definition is applied. Alternatively, the FEV_1_/FEV_6_ ratio adequately predicts airflow obstruction [8] but is less variable over time or across study sites [9]. Thus, defining airflow obstruction as FEV_1_/FEV_6_ below the 5th percentile or Lower Limit of Normal (LLN) can overcome these limitations [10].

**Figure 2:**
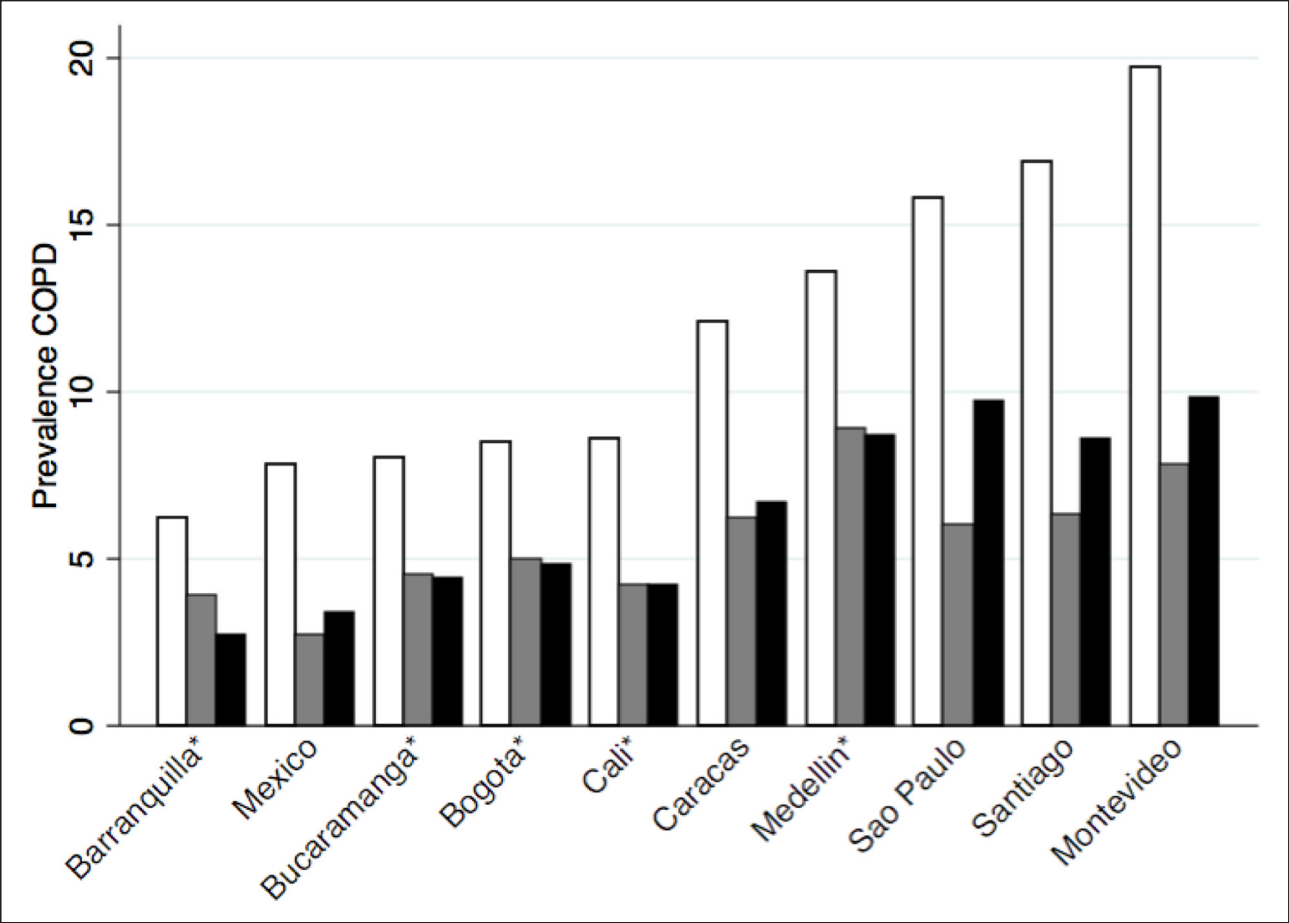
Prevalence of chronic obstructive pulmonary disease in Latin American cities by three spirometric definitions. Black bars LLN, white bars FEV_1_/FVC < 0.7 (global initiative for obstructive lung diseases), grey bars FEV_1_/FVC < 0.7 and FEV_1_ < 80% predicted (global initiative for obstructive lung diseases stages 2–4).

**Figure 3:**
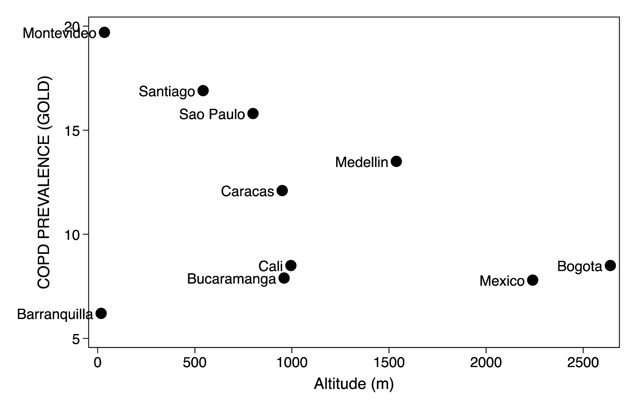
Prevalence of chronic obstructive pulmonary disease in Latin American cities and altitude above sea level. The unlabeled marker in the lower left extreme corresponds to Barranquilla and that in the right axis to Bogota, both in Colombia.

Table 1 and Figures 2 and 3 summarize data regarding COPD prevalence in the cities included in PLATINO and PREPOCOL. According to GOLD criteria, prevalence varied between 6.2% in Barranquilla, Colombia to 19.6% in Montevideo, Uruguay, both at sea level, but the latter, with an older population, mostly of European origin. As observed in other studies, prevalence increased with aging and male gender. The studies also documented the impact of performing spirometry without use of bronchodilators, a 30% increase in the prevalence of airflow obstruction [9]. Thus, it is important to base COPD prevalence estimates on the results of standardized spirometry conducted after bronchodilator use.

**Table 1:** Prevalence of COPD in Population-based Surveys in Latin America.

City	Altitude (metres above sea level)	Ever smokers (%)	Current smokers (%)	Cigarettes/day in smokers	Average pack-years in smokers	COPD (%)	COPD (GOLD 2–4) (%)	COPD (FEV1/FVC <LLN) (%)

Sao Paulo	800	56.7	24.0	15.4	24.5	15.8	6.0	9.7
Mexico	2240	43.8	25.3	6.0	10.3	7.8	2.7	3.4
Montevideo	35	57.4	28.4	15.3	27.6	19.7	7.8	9.8
Santiago	543	66.4	38.5	8.2	16.0	16.9	6.3	8.6
Caracas	950	57.7	28.5	10.5	18.9	12.1	6.2	6.7
Barranquilla*	18	45.0	13.9	8.9	14.4	6.2	3.9	2.7
Bogota*	2640	47.5	17.0	9.3	17.2	8.5	5.0	4.8
Bucaramanga*	960	43.2	13.0	8.5	15.3	8.0	4.5	4.4
Cali*	995	46.0	17.6	8.6	14.8	8.6	4.2	4.2
Medellin*	1538	60.5	29.8	11.9	21.2	13.6	8.9	8.7

COPD: Chronic Obstructive Pulmonary Disease, LLN: Lower Limit of Normal, the 5th percentile of gender-age and height expected values from a healthy population. *Obtained from the PREPOCOL study in Colombia, which used the turbine based Micro-loop, micro-medical spirometer. Other data from the PLATINO, based on measurements conducted with the ultrasonic based Easy-One spirometer.

These studies showed that underdiagnosis of COPD was common, with rates as high as 89% in some cities. Conversely, half of individuals with clinical diagnosis of COPD did not have evidence of airflow obstruction [11]. Both under- and overdiagnosis of COPD were attributed to lack of routine spirometry testing [11,12,13,14] and frequent reliance on respiratory symptoms to make clinical diagnosis of COPD [11]. Previous spirometry testing was very uncommon in PLATINO, even in individuals with a formal clinical indications for testing. Unfortunately, availability of spirometers is not sufficient as adequate implementation of lung function testing requires physician awareness and uptake [15].

Several associations with COPD have been reported in the PLATINO study.

Women in general, and those with airflow obstruction in particular, more commonly reported dyspnea, physical limitation, and fair to poor health status than men [16]. Individuals with COPD had more comorbidities, poorer health status [17], were more frequently underweight [18] and reported a negative impact in their ability to hold paying jobs [19]. Consistent with prior studies, COPD patients with more severe obstruction more frequently experienced exacerbations [20]. Individuals with the asthma-COPD overlap syndrome also had increased number of exacerbations and hospitalizations, lower lung function and more respiratory medications, therefore a greater health impact [21], although they tend to improve with inhaled corticosteroids.

## Screening for COPD

The potential benefits of early identification of COPD has been long debated. Such a strategy may be beneficial in Latin America given the high rate of underdiagnosis reported in population-based studies. A commonly proposed strategy for early identification has been to conduct spirometry testing among individuals at higher risk for COPD (smokers, particularly those with respiratory symptoms) [22]. An ongoing multicenter trial is assessing the value of COPD screening in primary care centers of Latin America [23,24,25,26]. The study confirmed a high frequency of underdiagnosis either using as criteria of airflow obstruction a post-bronchodilator FEV_1_/FVC ratio < 0.70 (77%) or by LLN (73%); overdiagnosis was high with 30% of subjects with a clinical diagnosis of COPD lacking objective evidence of airflow obstruction [26]. The prevalence of the asthma-COPD overlap syndrome represented between 11–26% of the COPD population depending on the definition used [24].

A study using data from the PLATINO and BOLD studies showed that a >70% predicted peak expiratory flow rate (PEFR), a simple low-cost test commonly used for asthma monitoring, could rule out severe airflow obstruction (GOLD stages 3–4) [27]. However, PEFR were measured during formal spirometry, and thus, potentially more precise than results obtained with simpler peak flow devices used in routine care. More recently, a population-based survey conducted in Mexico City, simultaneously performed standard spirometry and a six-second spirometry measurements using a simpler device called COPD-6. Although the simplified measurement demonstrated lower precision and reproducibility, the study showed that a more efficient screening can be achieved using a three-step strategy: 1) use a simple questionnaire to assess age and smoking history; 2) those at risk undergo simplified spirometry; 3) subject with FEV_1_/FEV_6_ < 80% are referred for full diagnostic spirometry [28]. This strategy leads to an 80% reduction in formal spirometry testing, a common barrier to COPD diagnosis. Additional advantages of the six-second spirometry include less complex training requirements, simplified quality control requirements and lower device cost, making it an ideal strategy for primary care settings, particularly in developing countries [15].

## Risk Factors for COPD in Latin America

As in other areas of the world, tobacco exposure has been consistently associated with COPD risk in Latin America. The state of the tobacco epidemic varies in different countries as well as in rural versus urban areas. The PLATINO survey showed a prevalence of current smoking ranging from 24% in Sao Paulo to 38% in Santiago de Chile. The mean number of cigarettes smoked also varied substantially from six cigarettes/day in Mexico City to 15 cigarettes/day in Sao Paulo; cumulative smoking history ranged from 10 pack-years in Mexico to 24 pack-years in Sao Paulo. In PLATINO, COPD prevalence in Mexico City was the lowest of all five cities as was the prevalence of tobacco consumption and the cumulative smoking exposure among smokers [29]. Fortunately, smoking trends have shown a declining pattern from 1980 to 2012 in most Latin American countries [30].

The potential contribution of alpha-1 antitrypsin deficiency was assessed by Perez Rubio et al. In Mexico, the frequency of PiS and PiZ variants of alpha-1 antitrypsin were very uncommon in Mestizo population [31], thus having a minimal contribution to the overall burden of COPD. However, a recent study showed that heterozygous genotypes were associated with lower lung function in smokers [31]. Polymorphisms of the matrix metalloproteinase (MMP) 2 and 9 [32] and tumor necrosis factor (TNF) promoters [33] have been associated with COPD in Mexican Mestizos. In other Latin American countries with a larger proportion of individuals with European descent, a discrete number of patients with severe deficiency have been identified.

Most COPD surveys show that 20–30% of individuals with airflow obstruction, regardless of the definition used, are never smokers, emphasizing important additional causes of airway obstruction. In the PLATINO survey, 33% of persons with COPD were never smokers [34], mostly elderly women with a physician diagnosis of asthma or previous tuberculosis [5,35]. Asthma is a well-recognized cause of reversible airflow obstruction and considered a different disease from COPD; thus, these patients should be excluded from COPD prevalence estimates. However, overlapping asthma and COPD is a well-established clinical entity that is being increasingly recognized [36]. Tuberculosis is associated with airflow obstruction and has been identified as a source of abnormal spirometry in the PLATINO and the PREPOCOL surveys [5,35] as well as other clinical studies [37]. These individuals have a different disease process and progression and thus, should not be combined with smoking-related cases of COPD.

**Figure 4:**
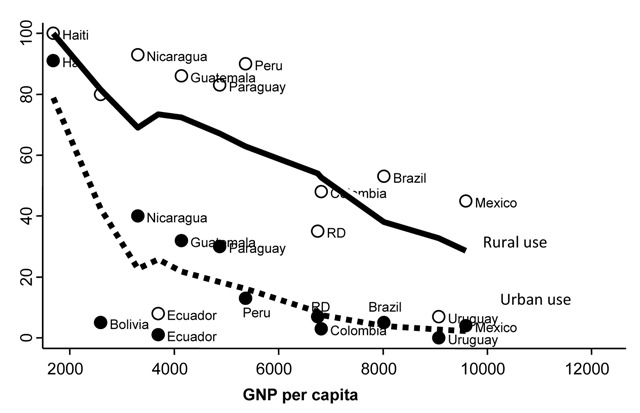
Dependence of biomass fuel use (vertical axis) on socioeconomic status (gross national income, horizontal axis) with a higher use in rural areas (empty circles) than in urban areas (filled circles). Solid fuel use has decreased in the last years but relationship remains similar. RD: Dominican Republic.

Biomass fuels are used for cooking and heating by nearly one half of the world population, mainly in underserved rural areas of developing countries (Figure 4) [38]. Current data shows that domestic exposure to biomass smoke is a risk factor for COPD [39,40,41,42,43,44,45,46]. The association between domestically inhaled biomass smoke has been long recognized [47,48] and considered a form of pneumoconiosis because of the consistent presence carbon deposits and fibrogenic dusts [44,49]. The risk estimates for COPD based on observational studies, including some carried out in Latin America, is 2–3 times higher in women [40,44,50] and approximately 1.8–1.9 higher in men exposed to biomass smoke [40,44,50]. While the association between exposure to biomass and respiratory symptoms is well demonstrated, the relationship with airflow obstruction has been more inconsistent. Longitudinal associations of lung function among biomass-exposed individuals were negative in a cohort study conducted in Mexico City [51]. However, in a longitudinal study in China, use of improved stoves was associated with a milder lung function decline [52]. If airflow obstruction is not due to an accelerated lung function decline, the adverse impact of biomass smoke inhalation may be related to changes in lung development and growth because of prenatal exposure during pregnancy.

Several studies have compared the characteristics of patients with COPD associated with biomass smoke exposure vs. tobacco smoking [51,53,54,55,56,57,58,59,60,61]. A recent review [55] concluded that, in general, persons exposed to biomass have mild or moderate airflow obstruction with normal diffusing capacity of carbon monoxide (DLCO) and absence of emphysema in computer tomography (CT) scanning [53,59,60,61]. In addition, higher rates of bronchial-hyperresponsiveness in biomass-exposed individuals than in smokers has been described [58], although these findings may represents a higher prevalence of asthma. Although CT scanning fails to show emphysema in non-smokers with COPD due to biomass smoke, autopsy series have found some degree of emphysema and small airways damage [54,62]. Additionally, pulmonary hypertension occurs in persons exposed to biomass smoke with COPD residing at moderate altitude in Latin America [48,54,62,63,64,65], and autopsy studies show that lung arteries of persons exposed to biomass smoke show more abnormalities in the intimal layer, than smokers with COPD [62].

## The Role and Impact of Hypoxemia

Lifelong exposure to altitude generates significant changes to lung development that may exert an impact on the risk for developing lung diseases. Interestingly, the altitude above sea level of cities participating in PLATINO study, showed a nearly perfect correlation with the adjusted prevalence of COPD (Figure 3); the higher the altitude, the lower the prevalence [66]. As mentioned previously, Mexico City, the city at the highest altitude, also had the lowest prevalence of tobacco use and the lowest rate of cumulative tobacco smoking (Table 1). Conversely, the PREPOCOL study, performed in a more homogeneous population than PLATINO, did not find differences in COPD prevalence based on altitude [5]. Thus, the impact of altitude on the risk of COPD remains undefined.

Hypoxemia significantly increases the risk of death in patients with COPD, as demonstrated in several studies [67]. Hypoxemia was also analyzed in the PLATINO survey in four cities [68]; the main determinants of a low oxygen saturation (≤88%) was altitude above sea level, age and body mass index. Thus, the prevalence of hypoxemia is expected to increase in Mexico City due to the combined effect of altitude, population aging and the obesity epidemic. Conversely, despite a high prevalence of airflow obstruction in Montevideo, a city near sea level, none of the obstructed individuals identified in PLATINO had low saturation of oxygen [68]. The same study also showed that only 8% of individuals in Mexico City with an oxygen saturation < 88% (6% of the studied population) were receiving oxygen, and conversely, half of those receiving oxygen had an saturation > 88% [68].

## Outcomes of Patients with COPD in Latin America

Currently, only PLATINO measured population-based longitudinal outcomes of patients with COPD, although unfortunately, these data are limited to three of the five cities originally studied [69]. The main causes of death in these patients were cardiovascular, respiratory and cancer, and risk of overall mortality was increased with COPD (hazard ratio 1.43 for FEV_1_/FVC < LLN and 2.0 for GOLD stages 2–4) [70].

In a cohort of patients living in Mexico City, survival was similar among COPD patients exposed to biomass smoke or tobacco once FEV_1_ was taken into account [56]. Smokers were typically more obstructed, whereas women exposed to biomass smoke were older, had a higher body mass index, had more hypoxemia and were more likely to live in a rural area [56]. Health-related quality of life was similarly abnormal in both groups. Lung function decline in women exposed to biomass smoke with a diagnosis of COPD in the same cohort was on average 21 mL/year, with no patient showing a decline > 40 mL/year [51], confirming the notion that accelerated decline in lung function is uncommon in these patients [71].

## COPD Costs

In all countries that collect cost data, COPD is one of the costliest diseases, particularly among patients with severe disease and those hospitalized in intensive care. Some of the most comprehensive assessments of the cost of COPD care was conducted as part of estimating the health costs of tobacco smoking in Mexico and other countries [72]; adding from 6 to 14% to personal health costs [72]. COPD-related health care costs at the Mexican National Institute of Social Security (IMSS), insuring workers and their families and covering nearly half of the Mexican population, were 1,469 million pesos in 2004 (about 157 million US dollars), 12.1% of the medical care costs for that year [73]. Estimates of COPD costs have been published from Colombia (4,600 million US dollars in 2004) [74] and of comparative costs of oxygen use in Chile, demonstrating in a small group of patients with COPD receiving home oxygen, that costs do not increase compared with a similar group of patients in a waiting list [75]. Limited information from Latin America is consistent with what has been found in developed countries: health expenditures in COPD care are extremely large.

## Treatment of COPD in Latin America

Although up-to-date guidelines for COPD treatment are available from ALAT [76,77,78] and from most national societies, the PLATINO survey showed highly variable and frequently suboptimal treatment of patients with COPD [79,80]. Only one half of smokers with COPD were counselled by a physician, and only a fourth were receiving any respiratory medication, often oral, and influenza vaccination was very scarce in several countries [79,80]. Additionally, 50% of individuals receiving bronchodilators did not demonstrate airflow obstruction on spirometry [79,80]. It is well known that availability of clinical guidelines, even if adoption is required by public health authorities, does not lead to a rapid change in physician practices. Thus, well planned and properly funded strategies should be implemented to improve management and outcomes of COPD patients in Latin America.

## COPD Prevention

Effective measures to prevent COPD in Latin America, as in other places, are primarily focused on anti-tobacco policies, which have fortunately expanded rapidly in most countries, particularly in Uruguay and Brazil. Reduction of exposures to occupational risks is also important but usually variable across Latin American countries and often suboptimal.

In many countries, great efforts have targeted a reduction in indoor biomass smoke exposure by shifting from the traditional highly inefficient and polluting three-stone stoves to improved and vented wood stoves. In some programs, millions of stoves have been built or purchased without solid evidence that it use leads to improved health or fuel economy [81,82]. Moreover, improved stoves are commonly abandoned due to a variety of reasons including a lack of risk perception among exposed individuals. In Michoacan, Mexico, a controlled intervention trial compared the impact of changing from a traditional open fire to an improved biomass stove [83]. When the trial was analyzed as intention to treat, no difference in outcomes was observed among women that changed the stove one year apart [83]. However, the results were confounded by the fact that up to one half of women in the intervention group returned to use the traditional open fire or utilized the improved stove in conjunction with the open fire. When actual exposure was assessed, those mainly using the improved stove had fewer respiratory symptoms [83] and a reduction of the FEV_1_ decline [83].

## A Primary Care Approach to COPD in Latin America

Given the high prevalence of COPD in Latin America and low rates of diagnosis and optimal treatment, there is a need for more efficient management of the disease. Primary care physicians are critical for early diagnosis and initial management of these patients. Specialists are needed to provide advanced care to patients with more severe disease or increased complexity due to comorbidities.

The results of the PLATINO survey suggest that primary care physicians may require additional training to better manage COPD patients in Latin America [84,85]. Integrated programs, such as the Practical Approach to Lung Disease [86,87,88,89], may help manage COPD as well as other respiratory conditions such as acute respiratory infections, pneumonia, tuberculosis and others depending on the country or region. As symptoms for many different respiratory diseases overlap, integrated programs may be particularly beneficial for countries with limited resources, and preferred to strategies only devoted to COPD [90]. Unfortunately, most countries often have implemented programs focused on individual diseases [91], even though an integrated primary care has been promoted for > 30 years [92].

## Research Opportunities

Many aspects of COPD in Latin America require further investigation. The prevalence of COPD has been explored in urban areas, but information from rural areas is scarce [83,93]. While a proportion of city inhabitants may have recently migrated from rural areas, they are usually a biased sample of those remaining in rural settings. Although a variety of studies have evaluated the mechanisms implicated in the association of biomass smoke exposure and COPD, more data is needed to develop effective interventions [94,95,96,97,98].

Additionally, adequately powered longitudinal studies are needed to explore the impact of biomass smoke exposure during pregnancy and early infancy on the risk of COPD. The currently recommended treatment of COPD associated with biomass smoke is usually the same as for tobacco-related disease [99]; however, the effectiveness of these interventions has not been documented in clinical trials.

In summary, COPD in Latin America is a leading health problem, requiring an integrated approach, and with numerous knowledge gaps. Prevention is a priority, including strong policies against tobacco smoking and indoor air pollution. Primary care of COPD should be emphasized, with efficient diagnostic strategies and better access to effective medications.
